# Invasive Group B *Streptococcus* Infections in Adults, England, 2015–2016

**DOI:** 10.3201/eid2606.191141

**Published:** 2020-06

**Authors:** Simon M. Collin, Nandini Shetty, Theresa Lamagni

**Affiliations:** National Infection Service, Public Health England, London, UK

**Keywords:** *Streptococcus agalactiae*, group B Streptococcus, invasive infection, epidemiology, adult, bacteria, England, streptococci

## Abstract

During 2015–2016, a total of 3,156 episodes of invasive group B *Streptococcus* (iGBS) infection in adults (>15 years of age) were recorded in England, corresponding to an annual incidence of 3.48/100,000 population. iGBS incidence was highest in older patients and women of childbearing age. The 493 pregnancy-related iGBS episodes correspond to a rate of 1.34/10,000 live births. In adults up to 60–69 years of age and in pregnant women, iGBS incidence increased with higher levels of socioeconomic deprivation. Hospital admissions associated with iGBS were predominantly emergency admissions (73% [2,260/3,099]); only 7% of nonpregnancy iGBS diagnoses were made >48 hours after admission. Underlying conditions were highly prevalent in nonpregnant adult case-patients, including cardiovascular (57%), lung (43%), and kidney (45%) disease and diabetes (40%). Post-iGBS episode 30-day and 12-month all-cause mortality rates in nonpregnant adults were 12% and 24%, respectively. No pregnancy-related iGBS deaths were identified.

*Streptococcus agalactiae* (group B *Streptococcus*; GBS) is implicated in a range of clinical manifestations in adults, including surgical site, skin and soft tissue, and urinary tract infections (*1*–*3*). Invasive GBS (iGBS) disease in adults is of growing clinical and public health concern (*4*–*6*), with incidence in England and Wales during 1996–2010 increasing almost 3-fold (*7*). The increasing prevalence of known risk factors for iGBS disease, including old age and underlying conditions such as diabetes (*8*–*11*), means that considerable healthcare, economic, and social costs will be associated with iGBS disease in adults. The advent of vaccines to prevent neonatal GBS disease raises the possibility of preventing iGBS disease in other patient groups (*12*). However, although neonatal iGBS has been the subject of epidemiologic research for several decades (*13*), adult iGBS disease is less well characterized.

The aim of this study was to characterize iGBS disease in adults by creating a national retrospective cohort of cases in England identified through laboratory surveillance linked to hospital admissions data. Our objectives were to describe characteristics of adults who received diagnoses of iGBS; time to infection, medical specialty, and healthcare resource use in patients admitted to a hospital within 7 days of diagnosis; concurrent conditions and surgery in current and prior admissions up to 1 year before diagnosis; and all-cause mortality within 30 days and within 1 year of an iGBS episode.

## Methods

### Case Definition

We defined iGBS infection through the isolation of *S. agalactiae* recorded in specimens from a normally sterile site, such as blood, cerebrospinal fluid, joint fluid, bone, pleural fluid, bronchoalveolar lavage, ascitic fluid, lymph node biopsy/aspirate, pericardial fluid, heart valve, brain abscess, or other organs. We considered laboratory test result records within 14 days to be part of the same episode and merged them accordingly.

### PHE Second Generation Surveillance System 

We extracted laboratory-confirmed cases of iGBS infection diagnosed from specimens taken during January 1, 2015–December 31, 2016 from the Public Health England (PHE) Second Generation Surveillance System (SGSS), the national communicable disease surveillance database, for analysis. SGSS collates microbiological diagnoses from laboratories across England (including all National Health Service [NHS] laboratories), primarily through automated upload (*14*–*16*). In addition to information about the laboratory, specimen, test method, and type of infection, SGSS records include patient identifiers and demographic data (name, sex, date of birth, and the patient’s NHS number).

### Hospital Episode Statistics 

We identified corresponding hospital admissions for episodes of iGBS infection through record linkage to Hospital Episode Statistics (HES; https://digital.nhs.uk). HES includes all NHS secondary care activity (98% of all hospital activity in the country [*17*]) that requires a hospital bed, including emergency and planned (elective) admissions, day cases, and births. HES admitted patient care data are collated by NHS Digital to reimburse NHS hospitals for the costs of care (using Healthcare Resource Group codes to group diagnoses and interventions). These data provide clinical, demographic and organizational information, including data on diagnoses and procedures, concurrent conditions (by code from the International Classification of Diseases, 10th Revision [ICD-10]), dates of admission, admission method (emergency or elective), care provider, and geographic variables mapped from a patient’s postcode.

### Data Linkage and Statistical Analysis

Our analysis of hospital admissions associated with iGBS episodes used a dataset created by linking SGSS laboratory records corresponding to iGBS cases with HES hospital admission records (using NHS numbers). Before performing this linkage, we sent patient identifiable data from SGSS records to NHS Digital’s Demographic Batch Service to validate recorded NHS numbers, trace missing patient NHS numbers, and obtain dates of death (*18*). We extracted corresponding HES records for a period 1 calendar year before and after the SGSS surveillance period, January 1, 2014–December 31, 2017. To analyze treatment specialties and Healthcare Resource Group when an iGBS episode had >1 associated admission, we selected the admission that was closest to the specimen request date; each admission could be represented by >1 treatment specialty. For each iGBS episode recorded in SGSS, we defined an associated hospital admission as an admission occurring within 7 days of the iGBS specimen request date (proxy for date of diagnosis). Pregnancy-related iGBS episodes were identified by an ICD-10 O or Z370 code for diagnosis at admission. We built multivariable models for risk factors associated with all-cause 30-day and 12-month mortality after iGBS unrelated to pregnancy, including age, sex, socioeconomic deprivation, underlying conditions, and type of hospital admission as a priori risk factors. We performed all analyses in Stata (https://www.stata.com).

## Results

**Figure 1:**
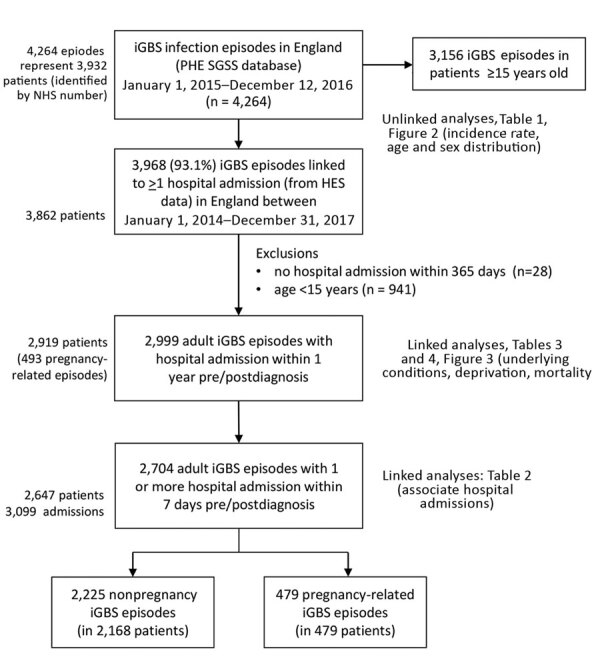
Data flowchart of iGBS infection in England, 2015 and 2016. iGBS, invasive group B *Streptococcus*.

During 2015–2016, a total of 3,156 iGBS episodes in adults (>15 years of age) were recorded in England, corresponding to an incidence rate of 3.48 per 100,000 population per year (Table 1). In 87% (2,753) of recorded episodes, GBS was isolated from a blood culture; the 3 next most common specimen types were from joint (5.6%), bone (2.7%), and bronchoalveolar lavage (2.4%). Of the 3,156 iGBS episodes, 2,999 (95.0%), representing 2,919 patients, could be linked to a hospital admission record within 1 year of iGBS diagnosis and 2,704 (representing 2,647 patients) to an admission within 7 days of iGBS diagnosis (associated hospital admission); of these 2,704 episodes, 479 (17.7%) were pregnancy related (Figure 1). Subtracting this proportion of cases from the 3,156 episodes for England during 2015–2016 gives an estimated incidence rate for iGBS in nonpregnant adults of 2.9/100,000 population/year.

**Table 1:** Numbers of patients and episodes of iGBS infection among adults in England, 2015–2016*

Age, y	No. iGBS episodes	No. patients	Male sex, no. (%)	No. (%) patients with >2 iGBS episodes†	No. (%) iGBS episodes per patient			Annual iGBS incidence‡
1	2	>3
15–19	21	21	7 (33.3)	0 (0.0)	21 (100.0)	0	0	0.33
20–29	241	230	27 (11.7)	3 (1.3)	227 (98.7)	2 (0.9)	1 (0.4)	1.63
30–39	388	376	58 (15.4)	2 (0.5)	374 (99.5)	2 (0.5)	0	2.66
40–49	277	261	128 (49.0)	3 (1.1)	258 (98.9)	2 (0.8)	1 (0.4)	1.87
50–59	363	334	205 (61.4)	13 (3.9)	321 (96.1)	9 (2.7)	4 (1.2)	2.50
60–69	493	466	278 (59.7)	11 (2.4)	455 (97.6)	8 (1.7)	3 (0.6)	4.13
70–79	565	533	304 (57.0)	18 (3.4)	515 (96.6)	17 (3.2)	1 (0.2)	6.76
>80	808	772	357 (46.2)	22 (2.8)	750 (97.2)	18 (2.3)	4 (0.5)	15.1
Total	3,156	2,993	1,364 (45.6)	72 (2.4)	2,921 (97.6)	58 (1.9)	14 (0.5)	3.48

*iGBS, invasive group B *Streptococcus*. †Recurrent iGBS episodes detected during Jan 1, 2015–Dec 31, 2016 based on iGBS episodes for which patient’s NHS number was recorded in PHE laboratory surveillance system (3084/3156 episodes) ‡Per 100,000 population. Numerator = iGBS episodes/2; denominator = Office for National Statistics mid-2016 population estimates for England (http://www.ons.gov.uk/peoplepopulationandcommunity/populationandmigration/populationestimates/datasets/populationestimatesforukenglandandwalesscotlandandnorthernireland)

### Characteristics of Adults’ iGBS Infection

**Figure 2:**
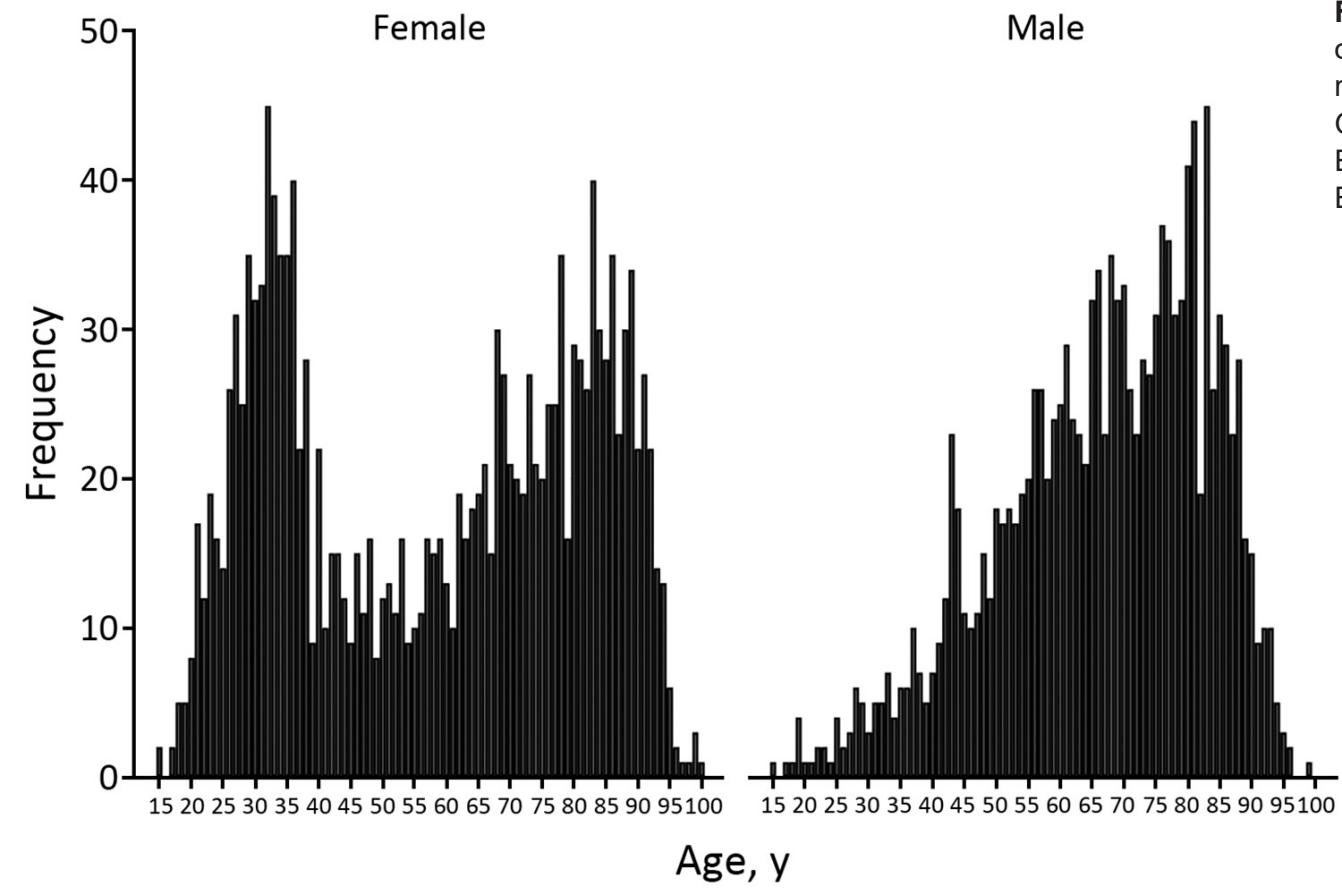
Age and sex distribution of patients >15 years of age who received diagnoses of invasive GBS infection, Public Health England laboratory surveillance, England, 2015 and 2016.

**Figure 3:**
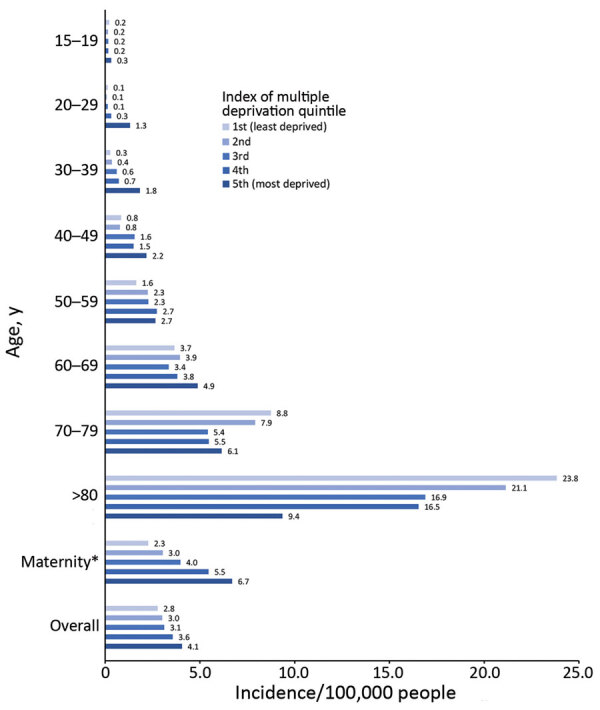
Social deprivation quintiles for patients >15 years of age who received diagnoses of invasive GBS infection, Public Health England laboratory surveillance, England, 2015 and 2016.

Analysis of sex and age identified that iGBS was most likely to occur in older patients and women of childbearing age (Figure 2). Most (97.6%; 2,921/2,993) patients had a single episode; 1.9% (58) had 2 and 0.5% (14) had >3 episodes (Table 1). There were 493 pregnancy-related iGBS episodes (1.34/10,000 live births). Assessment of incidence (excluding maternity cases) by socioeconomic deprivation identified higher rates in more deprived socioeconomic groups in all but the oldest (>70 years) age groups (Figure 3; Appendix Table 1). This social gradient was reversed in elderly patients, with adults >80 years of age in the least deprived quintile having a 2.5-fold higher iGBS rate than in the most deprived quintile (23.8 vs. 9.4/100,000 population), an effect that was more pronounced in male than in female patients (Appendix Table 2). Race and ethnicity was broadly representative of the population of England (Appendix Table 3). The proportion who were black (4.0%) was slightly higher than the national figure (3.3%), and the proportion who were mixed race was lower (0.8% vs. 2.2%).

### Hospital Admission, Time to Diagnosis, Medical Specialty, and Healthcare Resource Group

Associated admissions (within 7 days of diagnosis) were predominantly through the emergency department (2,260/3,099; 72.9%), except in women 20–39 years of age, for whom most admissions were classified as maternity-related (493/587; 84.0%) (Table 2). In nonpregnancy episodes, most iGBS diagnoses (2,064/2,225; 92.8%) were from specimens taken within 48 hours of admission, suggesting that the infection was community rather than healthcare associated. In pregnancy-related episodes, 37.4% (179/479) of diagnoses were made >2 days after admission (Appendix Table 4). Patients were under the care of 57 different medical specialties (Appendix Table 5); general medicine was the most common specialty (1,435/4,011; 35.8%), followed by geriatric medicine (10.9%) and obstetrics (10.9%). Of specialties with >50 associated admissions, the second highest proportion of iGBS diagnosed >2 days after admission, after obstetrics and gynecology (37.4%), was in urology (25.7%). The top 4 Healthcare Resource Group subchapters were obstetric medicine (451/2,704; 16.7%), infectious diseases and immune system disorders (431/2,704; 15.9%), respiratory system procedures and disorders (366/2,704; 13.5%), and skin disorders (319/2,704; 11.8%) (Appendix Table 6).

**Table 2:** Hospital admissions among adult patients with iGBS infection in England, 2015–2016*

Age, y	Male patients, no. (%)				Female patients, no. (%)			
	No. admissions	Elective	Emergency		No. admissions	Elective	Emergency	Maternity
15–19	4	2 (50.0)	2 (50.0)		14	0	6 (42.9)	8 (57.1)
20–29	29	2 (6.9)	27 (93.1)		237	5 (2.1)	27 (11.4)	205 (86.5)
30–39	54	2 (3.7)	50 (92.6)		350	10 (2.9)	51 (14.6)	288 (82.3)
40–49	121	9 (7.4)	109 (90.1)		140	12 (8.6)	81 (57.9)	46 (32.9)
50–59	204	30 (14.7)	170 (83.3)		140	34 (24.3)	105 (75.0)	1 (0.7)
60–69	292	35 (12.0)	250 (85.6)		197	25 (12.7)	171 (86.8)	0
70–79	307	23 (7.5)	283 (92.2)		230	16 (7.0)	210 (91.3)	0
>80	366	26 (7.1)	331 (90.4)		414	19 (4.6)	387 (93.5)	0
Total	1,377	129 (9.4)	1,222 (88.7)		1,722	121 (7.0)	1,038 (60.3)	548 (31.8)

*N = 3,099 associated hospital admissions (within 7 d of specimen date) represent 2,704 invasive GBS episodes in 2,647 patients. iGBS, invasive group B *Streptococcus*.

### Underlying Conditions and Surgery <1 Year before iGBS Diagnosis

Analysis of ICD-10 codes recorded in the current and previous (<1 year pre-iGBS) hospital admissions identified underlying conditions as common in nonpregnant adults with iGBS disease (Table 3). Cardiovascular disease was recorded for 57.5% (1,440/2,506) of all nonpregnancy iGBS episodes, lung disease for 42.9% (1,076/2,506), kidney disease for 44.5% (1,115/2,506), and diabetes for 39.9% (1,000/2,506). Prevalences of underlying conditions were much lower in pregnancy-related cases; most (349/493; 70.8%) had no recorded underlying condition (Appendix Table 7). After diagnostic imaging, testing, and rehabilitation (1,674/2,999; 55.8%), the 4 most commonly recorded current or prior medical procedures by anatomic chapter were arteries and veins (550/2,999; 18.3%); pregnancy, childbirth, and the puerperium (470/2,999; 15.7%); bones and joints (439/2,999; 14.6%); and urinary (433/2,999; 14.4%) (Appendix Table 8).

**Table 3:** Underlying conditions among adult patients with invasive GBS (iGBS) infection unrelated to pregnancy in England, 2015–2016*

Conditions	Age, y								Total
	15–19	20–29	30–39	40–49	50–59	60–69	70–79	>80	
No. iGBS episodes	12	49	98	204	339	474	545	785	2,506
Diabetes	1 (8.3)	5 (10.2)	17 (17.4)	62 (30.4)	162 (47.8)	212 (44.7)	267 (49.0)	274 (34.9)	1,000 (39.7)
Peripheral vascular disease	1 (8.3)	5 (10.2)	31 (31.6)	46 (22.6)	113 (33.3)	154 (32.5)	203 (37.3)	289 (36.8)	842 (33.6)
Cancer	2 (16.7)	2 (4.1)	1 (1.0)	28 (13.7)	57 (16.8)	77 (16.2)	107 (19.6)	163 (20.8)	437 (17.4)
Cardiovascular disease	3 (25.0)	12 (24.5)	32 (32.7)	58 (28.4)	125 (36.9)	249 (52.5)	355 (65.1)	606 (77.2)	1,440 (57.5)
Lung disease	5 (41.7)	20 (40.8)	39 (39.8)	72 (35.3)	148 (43.7)	193 (40.7)	242 (44.4)	357 (45.5)	1,076 (42.9)
Kidney disease	2 (16.7)	9 (18.4)	19 (19.4)	69 (33.8)	117 (34.5)	197 (41.6)	268 (49.2)	434 (55.3)	1,115 (44.5)
Neurologic disorders	2 (16.7)	11 (22.5)	21 (21.4)	53 (26.0)	111 (32.7)	124 (26.2)	167 (30.6)	311 (39.6)	800 (31.9)
Arthritis	0	5 (10.2)	12 (12.2)	42 (20.6)	74 (21.8)	125 (26.4)	189 (34.7)	294 (37.5)	741 (29.6)
None of these	3 (25.0)	12 (24.5)	23 (23.5)	28 (13.7)	27 (8.0)	39 (8.2)	17 (3.1)	15 (1.9)	164 (6.5)

*Values are no. (%) patients. Predefined conditions (Appendix Table 7) identified in current and prior admissions (up to 1 y prediagnosis). iGBS, invasive group B *Streptococcus*.

### All-Cause Mortality after iGBS

Overall all-cause mortality in nonpregnant adults in the 30 days after an iGBS episode was 12.5% (313/2,506) (Table 4; Appendix Table 9). With extension to the 12 months after diagnosis, all-cause mortality increased to 29.2% (731/2,506). There were no deaths related to the 493 maternal iGBS episodes. For iGBS unrelated to pregnancy, 30-day mortality after an emergency admission was 13.3% (306/2,299), compared with 3.4% (7/206) for elective admissions (p<0.001). In a multivariable model, the strongest predictors of 30-day all-cause mortality for iGBS unrelated to pregnancy were older age (odds ratio [OR] 3.7 [95% CI 1.5–9.3] for age >80 years compared with 20–29 years), concurrent conditions (OR 5.3 [95% CI 1.7–16.9] for any of the conditions shown in Table 3 versus none), and emergency admission (OR 3.4 [95% CI 1.6–7.5] versus nonemergency admission) (Appendix Table 10). Estimates suggested an increased risk of death with higher levels of social deprivation, but these apparent effects were not supported by statistical evidence. In relation to concurrent conditions recorded in the current or prior hospital admission(s), we observed the highest 30-day mortality for iGBS unrelated to pregnancy in patients with kidney disease (221/1,115; 19.8%), followed by cancer (81/437; 18.5%), lung disease (186/1,076; 17.3%), and neurologic disorders (138/800; 17.3%) (Appendix Table 11). We observed similar effects for 12-month mortality (Appendix Tables 12, 13).

**Table 4:** All-cause 30-day and 12-month mortality among adult patients with iGBS infection in England, 2015–2016*

Age, y	Male patients					Female patients, excluding pregnancy-related episodes			
	No. iGBS episodes	All-cause deaths within 30 d, no. (%)	All-cause deaths within 1 y, no. (%)	Median (IQR) time to death, d		No. iGBS episodes	All-cause deaths within 30 d, no. (%)	All-cause deaths within 1 y, no. (%)	Median (IQR) time to death, d
15–19	6	0	3 (50.0)	39 (33–170)		6	2 (33.3)	2 (33.3)	4 (2–6)
20–29	27	1 (3.7)	2 (7.4)	144 (2–285)		22	0 (0.0)	2 (9.1)	179 (33–325)
30–39	59	4 (6.8)	7 (11.9)	3 (1–159)		39	1 (2.6)	2 (5.1)	58 (2–113)
40–49	120	14 (11.7)	27 (22.5)	26 (2–68)		84	3 (3.6)	8 (9.5)	37 (15–124)
50–59	212	21 (9.9)	44 (20.8)	39 (12–135)		127	3 (2.4)	15 (11.8)	80 (40–177)
60–69	281	21 (7.5)	51 (18.1)	45 (11–168)		193	21 (10.9)	46 (23.8)	72 (3–177)
70–79	310	38 (12.3)	93 (30.0)	57 (13–145)		235	34 (14.5)	66 (28.1)	30 (8–119)
>80	359	77 (21.4)	177 (49.3)	41 (10–188)		426	73 (17.1)	186 (43.7)	54 (8–136)
Total	1,374	176 (12.8)	404 (29.4)	44 (10–167)		1,132	137 (12.1)	327 (28.9)	53 (8–138)

*iGBS, invasive group B *Streptococcus*; IQR, interquartile range.

## Discussion

We characterized cases of iGBS disease occurring in England over a 2-year period. The annual incidence rate of 3.48/100,000 population during 2015–2016 suggests a continuation of the trend in adults during 1991–2010, when incidence increased from 0.92 to 2.39/100,000 population (*7*). This trend is largely attributable to cases in adults, particularly the elderly. A prominent feature of the iGBS age distribution in England is the relatively higher incidence among women of childbearing age, as also seen in some other countries (*8*). Overall incidence in England for 2015–2016 (≈3/100,000 population, excluding maternity cases) was one third the rate in nonpregnant adults in the United States for the same period (*6*). This finding is the opposite of the reported rates for pregnancy-related iGBS, which are lower in the United States (0.1/1,000 live births) compared with England (0.3/1,000 live births) (*19*), possibly because antenatal GBS screening in the United States leads to widespread use of intrapartum antimicrobial prophylaxis.

Prevalence of underlying conditions appeared to be much higher in iGBS cases than in the adult population in England (*20*). For example, 40% of persons with iGBS had diabetes (compared with 7% in the general population), 17% cancer (2%), 58% cardiovascular disease (1%), 45% kidney disease (4%), and 30% arthritis (1%). These findings are consistent with other studies of iGBS in adults (*5*,*6*,*8*–*11*,*21*,*22*); the increasing prevalence of predisposing medical conditions in the adult population may account for rising rates of iGBS disease (*4*–*8*). Vaccination to prevent iGBS in adults could be a key component of future preventive measures if appropriately targeted. The cost-effectiveness of GBS vaccination has been evaluated (favorably) in relation to neonatal disease (*23*,*24*), but health economic studies encompassing nonpregnancy-associated adult disease are lacking (*25*). Health economic studies of GBS vaccination to prevent GBS disease in adults would need to take into account all manifestations of GBS disease, including noninvasive infections (*26*) and the possible prevention of antimicrobial resistance (*6*,*27*,*28*).

Further prevention measures could be identified through improved understanding of the role of healthcare interventions and the wider hospital environment in enabling disease occurrence (*29*). Although most of the cases in this study were diagnosed at admission, suggesting community acquisition, the high prevalence of underlying conditions and the numbers of patients having medical procedures in the preceding year suggest that prior healthcare exposure was likely for a sizable proportion of patients.

In our study, 30-day mortality (12%) was higher than the case-fatality rate reported recently from a population-based study in the United States (6%) (*6*), but we included all-cause deaths within 30 days of iGBS rather than deaths occurring during hospitalization for iGBS. The high 12-month mortality rate that we observed in the youngest (15–19 years) age group (5/12 cases) reflected deaths in young adults with other medical conditions (2 with cancer, 1 with cardiovascular disease, 1 with multiple concurrent conditions).

The steep gradients observed over quintiles of social deprivation, with more deprived quintiles having higher iGBS incidence up to age 60–69 years but lower incidence thereafter, could be attributed partly to concurrent conditions, although overall prevalence of such conditions did not differ substantially across the quintiles. Social gradients have been reported for different infectious diseases in England (*30*), including incidence increasing with deprivation for invasive group A *Streptococcus*, meningococcal, and pneumococcal infection. Incidence of community-acquired pneumonia in older (>65 years of age) adults in the United Kingdom showed a steep increase with level of deprivation (*31*), the opposite of the trend that we observed for iGBS in adults >70 years of age.

The trend that we observed of increasing maternity-related iGBS incidence with higher levels of social deprivation is entirely consistent with the trend reported for risk of severe maternal sepsis in the United Kingdom (*32*). The potential for iGBS prevention in this group is supported by a study showing that >70% of women across all social grades would be likely to accept antenatal GBS vaccination (*33*).

Although PHE’s microbiology surveillance database has national coverage (*34*), reporting of GBS infection is not mandatory, and the completeness of reporting is unknown. A comparison of MRSA and *E. coli* data reported to a separate mandatory surveillance system showed that voluntary reporting represented >80% of records for bacteremia caused by these 2 species (*35*,*36*). A further limitation of our analysis is that, even with linkage to hospital episode data, we lacked sufficiently detailed clinical information on the manifestations and severity of the iGBS cases and outcomes resulting from GBS. This inability to ascertain clinical case characteristics constrained us to analyzing iGBS patients as a homogeneous group, which they will not be in practice (*2*,*5*,*6*,*9*,*37*). Although bronchoalveolar lavage is not universally considered a sterile site sample, we included these specimens in our iGBS case definition to capture lower respiratory tract infections. Although we cannot completely discount the possibility that some of the respiratory isolates were contaminants, national standards for microbiology laboratories provide guidelines to differentiate these from clinically significant infections (*38*).

In conclusion, incidence of iGBS disease in adults continues to increase in England, and high risk groups include the elderly, pregnant women, and adults with underlying conditions. Most nonpregnant adults have emergency cases, and all-cause mortality is high (one quarter of cases). Reasons for the strong social gradients at different ages merit further investigation, as does the potential for prevention of iGBS through vaccination.

Appendix Additional information on the study of invasive group B *Streptococcus* infections among adults, England, 2015–2016.
