# Correction: Vol. 26, No. 6

**DOI:** 10.3201/eid2709.C12709

**Published:** 2021-09

**Keywords:** errata, erratum, correction

The rate of pregnancy-related invasive group B *Streptococcus* episodes was misstated in Invasive Group B *Streptococcus* Infections in Adults, England, 2015–2016 (S.M. Collins et al.). The correct rate is 4.09/10,000 live births. The article has been corrected online (https://wwwnc.cdc.gov/eid/article/26/6/19-1141_article).

